# Determining Factors in the Therapeutic Success of Checkpoint Immunotherapies against PD-L1 in Breast Cancer: A Focus on Epithelial-Mesenchymal Transition Activation

**DOI:** 10.1155/2021/6668573

**Published:** 2021-01-07

**Authors:** Mariana Segovia-Mendoza, Susana Romero-Garcia, Cristina Lemini, Heriberto Prado-Garcia

**Affiliations:** ^1^Departamento de Farmacología, Facultad de Medicina, Universidad Nacional Autónoma de México, Coyoacán, 04510 Ciudad de México, Mexico; ^2^Laboratorio de Onco-Inmunobiología, Departamento de Enfermedades Crónico-Degenerativas, Instituto Nacional de Enfermedades Respiratorias, “Ismael Cosio Villegas” Calzada de Tlalpan 4502, Col. Sección XVI, Ciudad de México 14080, Mexico

## Abstract

Breast cancer is the most common neoplasm diagnosed in women around the world. Checkpoint inhibitors, targeting the programmed death receptor-1 or ligand-1 (PD-1/PD-L1) axis, have dramatically changed the outcome of cancer treatment. These therapies have been recently considered as alternatives for treatment of breast cancers, in particular those with the triple-negative phenotype (TNBC). A further understanding of the regulatory mechanisms of PD-L1 expression is required to increase the benefit of PD-L1/PD-1 checkpoint immunotherapy in breast cancer patients. In this review, we will compile the most recent studies evaluating PD-1/PD-L1 checkpoint inhibitors in breast cancer. We review factors that determine the therapeutic success of PD-1/PD-L1 immunotherapies in this pathology. In particular, we focus on pathways that interconnect the epithelial-mesenchymal transition (EMT) with regulation of PD-L1 expression. We also discuss the relationship between cellular metabolic pathways and PD-L1 expression that are involved in the promotion of resistance in TNBC.

## 1. Introduction

Breast cancer is the most frequent female-associated neoplasm that affects women worldwide [1]. It can be defined as a set of biological and molecular heterogeneous diseases originating in the breast. Based on different clinicopathological characteristics, there are several types of breast cancer. According to the differential expression of estrogen receptor (ER), progesterone receptor (PR), and epidermal growth factor receptor type 2 (HER2), breast cancer has been traditionally classified into three different phenotypes: luminal (ER+/PR+), HER2+, and triple-negative breast cancer (TNBC) [2]. Within the TNBC phenotype, there are 6 subclasses characterized by the expression of different molecules [3, 4]. TNBC is known to be the most aggressive phenotype, with few therapeutic opportunities and with poor patient prognosis. The luminal and HER2+ phenotypes are treated with target therapies against ER and HER2 proteins [5–7].

Current evidence has established that the response to therapy and the prognosis of breast cancer patients may be conditioned by the intrinsic heterogeneity of breast tumors, especially in TNBC [8]. The profile of tumor-infiltrating lymphocytes (TILs) and how it is conformed within the tumor has received special attention because the profile of TILs is also considered a crucial factor that determines the therapeutic response of different chemotherapeutic agents even in nonimmune based therapies [9]. In fact, the proportion and type of immune cells infiltrating the tumor can vary according to the molecular phenotype of this pathology [10]. In addition, the generation of resistance to conventional therapies is a critical concern in the clinic, in which immunotherapy has been considered an alternative in recent years.

For all of the above, the employment of selective immune therapies has been an important clinical tool against cancer. More specifically, the use of immune-checkpoint inhibitors targeting the programmed death receptor-1/ligand-1 (PD-1/PD-L1) axis has dramatically changed the outcome for cancer patients [11, 12].

## 2. PD-1/PD-L1 Axis

Tumors are known to use several mechanisms to disrupt the function of tumor-specific T cells, macrophages, and other immune cells. Among these mechanisms are the expression of ligands which bind to inhibitory receptors expressed on T cells, suppressing their function. T cells are activated by the interaction of the TCR/CD3 complex with a specific antigen presented by the APC, and costimulatory signals mediated by molecules such as CD28. TCR engagement and CD28 costimulation promote phosphorylation of a wide array of molecules involved in transduction pathways that promote T cell activation [13]. Conversely, several coinhibitory molecules have also been discovered that regulate the immune system, as is the case for T cells. In particular, the checkpoint PD-1/PD-L1 axis is one of the best-known mechanisms that modulates the functioning of immune cells [14].

PD-1 is a transmembrane protein that belongs to the B7 family of immune costimulatory/inhibitory molecules, and it is commonly expressed on the surface of activated T and B lymphocytes, myeloid cells, and TILs [15, 16]. PD-1 has two known ligands, PD-L1 and PD-L2 [17, 18]. Different signaling pathways including NF-*κ*B, MAPK, PI3K, mTOR, and JAK/STAT have been shown to modulate the activation/expression of PD-1 as well as its ligands [19–21]. PD-1 induces its signaling pathway after T cell receptor- (TCR-) crosslinking. Upon TCR stimulation, the tyrosine residues of the immunoreceptor tyrosine-based switch motif (ITSM) on the cytoplasmic tail of PD-1 become phosphorylated, recruiting SHP-1 and SHP-2 phosphatases, which in turn dephosphorylate proximal signaling molecules downstream of the TCR, leading to a negative immunomodulation. The inhibition of the expression of transcription factors associated with effector cell function, including GATA3 (GATA Binding Protein 3), T-bet, and eomesodermin (Eomes) is promoted after TCR engagement and PD-1 interaction [22].

PD-L1 and PD-L2 show a different pattern of expression; while PD-L2 expression is limited to antigen-presenting cells (APCs), PD-L1 is expressed in wider arrays of tissues including APCs. Among the nonhematopoietic tissues that constitutively express PD-L1 are cells from immune privilege sites such as testes, cornea, placenta, and pancreatic islets. PD-L1 has been shown to act as a preponderant tumor evasion mechanism; therefore, the mechanisms that modulate its expression have been the subject of intense research [23]. Different articles have pointed out that amplification or mutations of important protooncogenes including Kirsten rat sarcoma viral oncogene homolog (KRAS), TP53, and hepatocyte growth factor receptor (HGFR or c-MET) are significantly associated with high levels of mRNA and protein expression of PD-L1 in lung cancer [24–26]. For instance, KRAS has been associated with the stabilization of AU-rich elements in the 3′ UTR region of PD-L1, as well as the reduction of the expression of genes related with the presentation of different antigens via MHC class I molecules to T cells [27]. Thus, antigen processing/presentation on immune cells is deregulated, which if joined to PD-L1 expression on cancer cells, results in a positive feedback that sustains the immune-resistant state [28].

PD-L1 expression is also subject to epigenetic modifications. In this sense, the regulation of the expression of PD-L1 has also been associated to the bromodomain extra terminal (BET) family in ovarian cancer [29]. The BET family consists of four different proteins (BRD2, BRD3, BRD4, and BRDT), which are implicated in the transcription modulation of some oncogenes through a bromodomain that recognizes promotor hystone lysine acetylation and recruits other transcription factors [30]. Recently, it was reported that BET proteins control the expression of PD-1 in activated T cells and PD-L1 in TNBC cells. In addition, these proteins also regulate IFN-*γ* secretion by activated T cells when they are cocultivated with TNBC cells [31, 32]. Different epigenetic changes including methylation and histone modification have been reported in the promoter of the PD-L1 gene in different types of cancer [33]. However, the role of these biological changes has been not deeply explored in breast cancer.

TNBC is also considered the most undifferentiated subtype within the variety of breast cancer. Interestingly, the number of gene copies of CD274 (the PD-L1 gene) has been found augmented in TNBC samples [34], which suggest that it is mostly feasible for TNBC tumors to develop immunoescape features. Several studies point out that the overexpression of PD-L1 is the result of a genomic amplification of chromosome 9p24.1, which encodes the PD-L1 gene [35]. The activation of Janus kinase 2 (JAK2)/STAT1 by the addition of IFN-*γ* to TNBC cell cultures has been implicated in the amplification of chromosome 9p24.1 [36]. The actions described above draw attention due to the fact that IFN-*γ* is a cytokine that usually participates in antitumor responses, although recent literature points out that IFN-*γ* has dual effects in both tumor escape or tumor promotion [37].

Furthermore, the interaction of PD-L1 to its receptor in conjunction with other costimulatory molecules in naïve CD4+ T cells promote *de novo* transformation to the regulatory T cell (Treg) phenotype, through the inhibition of the mammalian target of rapamycin- (mTOR-) Akt signaling cascade [38]. The actions described above favor cancer immune escape [39]. Of note, other costimulatory proteins including CD80, a protein expressed on APCs and activated T cells, interacts with PD-L1. This interaction impairs binding of PD-L1 to PD-1, subsequently abrogating PD-1-mediated T cell suppression, which may be decisive for inducing optimal antitumoral responses [40].

## 3. A Landscape for the Use of PD-1/PD-L1 Checkpoint Inhibitors in Breast Cancer

The blockade of the PD-1/PD-L1 axis as a means for treating cancer was discovered by James P. Allison and Tasuku Honjo, who won the Nobel Prize of Physiology of Medicine for this breakthrough in 2018 [41]. Nowadays, the blockade of these molecules has been shown to be one of the most successful immunotherapies focused on enhancing the activity of immune cells against tumor cells. The above is closely related to the loss of immunologic control that is considered as one of the hallmarks of cancer [42]. The employment of these types of therapies has been widely proved in melanoma and non-small-cell lung cancer (NSCLC). In fact, different blockers of PD-1/PD-L1 interaction have been approved by the Food and Drug Administration (FDA) [43, 44]. Current methods to achieve efficient inhibition of PD-1/PD-L1 proteins are based on anti-PD-1 or anti-PD-L1 antibodies, gene silencing, and small-molecule pathway inhibition [45, 46]. It has been revealed that the PD-L1 molecule has a broad distribution in cancer, being located at serum levels, on the cancer cell membrane, and at the cytoplasmic or nuclear level, which limits the therapeutic efficacy of antibodies against this target [45].

Recently, the blockade of PD-1/PD-L1 has been investigated in breast cancer. In fact, the pathophysiological role of the PD-1/PD-L1 pathway has also been demonstrated using *in vitro* breast cancer models. First, the expression of PD-L1 has been reported in breast cancer cell lines with different phenotypes. MCF-7 (ER+) and MBA-MB-231 (TN) cells exhibit high levels of this protein. The coincubation of both types of breast cancer cells with human T lymphocyte cells (Jurkat cell line) or peripheral blood mononuclear cells (PBMCs) results in the inhibition of T cell activation via reduction of the expression of nuclear factor of activated T cells (NFAT), as well as cytokine expression (IL-2 and IFN-*γ*) [47]. Of note, blocking PD-1/PD-L1 interaction with the inhibitor A0-L significantly restores the activation of T cells, in addition to the secretion of IFN-*γ* and IL-2 cytokines by PBMC cells [47].

Some clinic reports have mentioned that not all patients with breast cancer have a good outcome after treatment with PD-1/PD-L1 checkpoint immunotherapies [48]. Several reasons are associated with the poor effectiveness of immunotherapies in breast cancer patients, such as the type and activation grade of immune infiltrating cells, the tumor cytokine pattern within the microenvironment, tumor cell mutations, exposition to chemotherapeutic agents, and an imbalance of anti- or proapoptotic proteins, along with the molecular phenotype of breast cancer [49]. Of note, approaches using therapeutic blocking of PD-1/PD-L1 have obtained better clinical results in breast cancer patients with a TN phenotype as compared with the luminal and HER2 phenotypes. However, little is known about what factors are crucial for an optimal response to PD-1/PD-L1 therapies in breast cancer [50].

PD-L1 has been shown to be expressed on both the membrane and cytoplasm by breast tumor cells but not by adjacent normal tissues [51]. PD-L1 expression has been detected by different techniques in all breast cancer subtypes. Its expression has been associated with larger tumors and absence of hormone receptors. The histological origin of the tumor cells seems to be related to PD-L1 expression, as invasive lobular carcinomas have lower expression of this molecule compared with ductal carcinomas [52]. Breast cancer cells also express PD-L1, although at lower levels compared to the TN phenotype. Immunohistochemical analyses of breast cancer tissues considering positive cells for PD-L1 revealed that HER2-positive breast cancers also express PD-L1 protein, although they showed lower PD-L1 levels compared to the TN phenotype. Nevertheless, PD-L1 expression in both phenotypes of breast cancer cells correlated with poor patient survival in patients that previously received chemo-, radio-, or endocrine therapy. In general, higher PD-L1 expression has been observed in TNBCs as compared to non-TNBCs; its upregulation has also been associated with overall, metastasis-free survival, and pathological complete response after neoadjuvant chemotherapy [51, 53–55].

Another important correlation between the expression of PD-L1 in TNBC might be also associated with the grade of cell differentiation. Supporting this fact, expression of this protein has been found to be increased in metastatic lung cancer samples compared with primary lung cancer lesions [24]. A similar PD-L1 expression pattern was found in breast cancer. Primary neoplasm lesions or tumors in early stages do not express high levels of PD-L1 as compared with the most aggressive counterpart present in advanced stages, where cell dedifferentiation is extremely marked [56]. In addition, PD-L1 expression is greater in patients with TN phenotype, lymph node metastasis, advanced clinical staging (high TNM stage), high Ki-67 score, and histopathological grading [52, 57–61]. However, no statistically significant relationship between PD-L1 expression in tumors, or within the tumor microenvironment, and clinicopathological parameters such as age, tumor size, and tumor grade has been established [58].

In spite of several efforts to standardize the detection of PD-L1 as a biomarker, the search of the expression of PD-L1 in TNBC biopsies is a complex process due to the intertumoral heterogeneity in the expression of this molecule [62]. Besides tumor cells, the components of the stroma (e.g. fibroblasts, myofibroblasts, leukocytes, endothelial cells, macrophages, adipocytes, or extracellular matrix) might also express PD-L1 and play a role in inducing T cell dysfunction.

In this regard, the expression of PD-L1, as detected by immunohistochemical evaluation, has been directly related with a high grade of maturation of stroma, which can be defined by the cumulus of several mature collagen fibers into multiple layers. The mature stroma in conjunction with PD-L1 expression predicts breast cancer outcome. Moreover, the relationship between hormone receptor negative tumors and the higher frequency of positive stromal PD-L1 staining has been observed [63]. Thus, identification of tumor stromal type, governed by the maturation state of collagen fibers, might be incorporated into clinical routine for achieving an optimal therapeutic scheme in breast cancer patients [63].

Additionally, some clinical reports have also evaluated the expression of PD-L1 in different samples of breast cancer patients in conjunction with the TIL population [64]. Interestingly, TN breast tumors are considered as the phenotype with the highest immune infiltration as compared with the other phenotypes [65]. Of note, there is an immunomodulatory subtype within the TNBC, which probably has a greater response to immunotherapies. This could partly explain why PD-L1 inhibitors alone or in combination with other therapies have been predominantly studied in the TN phenotype [66–68]. Additionally, expression of PD-L1 by cells from the tumor microenvironment, as well as the number of TILs, have emerged as crucial determining factors for breast cancer therapy, impacting in patient survival [9, 64, 69]. PD-L1 promotes an altered function of T cells [70, 71]. Thus, we hypothesize that the majority of the immune cells inside the tumor might be rendered dysfunctional because breast cancer samples with a TN phenotype also display high levels of PD-L1.

## 4. PD-L1 Inhibitors

Here, we present different PD-1/PD-L1 inhibitors that have been tested in breast cancer (Table 1), as well as other tumors. We describe some of their molecular characteristics and immune-activation capacity in addition to presenting molecular markers associated with prognosis.

### 4.1. Atezolizumab

Atezolizumab is a humanized Fc*γ*R binding-deficient anti-PD-L1 antibody that was approved by the FDA as a first-line treatment for cisplatin-resistant metastatic urothelial carcinoma and for metastatic NSCLC [72, 73]. In addition, atezolizumab has also been approved by the FDA as second-line therapy for advanced bladder cancer [74]. Atezolizumab has shown clinical improvement in TNBC patients with metastatic disease, reflected as longer overall survival in monotherapy and combined with chemotherapy [75–77]. In fact, in March 2019, the FDA approved atezolizumab in combination with chemotherapy based on paclitaxel for the treatment of metastatic TNBC patients with positive PD-L1 protein expression [78]. Unlike other malignancies, in breast cancer, some molecular markers have been recently associated to the patient's response to atezolizumab. In this regard, serum levels of the lactate dehydrogenase (LDH), a metabolic enzyme that converts pyruvate to lactate, are associated with reduced clinical benefit in breast cancer patients treated with atezolizumab monotherapy [22, 76]. This fact is relevant because, although atezolizumab may be able to inhibit the PD-L1 protein and activate the immune response, metabolic reprogramming of tumor cells will be a predominant way for cancer survival, as it has been established before in different types of cancer [79].

### 4.2. Avelumab

Avelumab is a human monoclonal antibody that targets PD-L1. It is approved by the FDA for the treatment of metastatic Merkel cell carcinoma (skin cancer) and advanced urothelial carcinoma [80, 81]. Avelumab can activate both adaptive and innate immune mechanisms to destroy cancer cells, unlike other immune therapies directed against the PD-1/PD-L1 axis [82]. Avelumab has been shown to block PD-1/PD-L1 interaction, as well as to activate natural killer (NK) cells through a mechanism known as tumor-directed antibody-dependent cell-mediated cytotoxicity (ADCC). Thus, ADCC is thought to be promoted by the crystallizable fragment (Fc) region belonging to avelumab, which engages with the Fc*γ* receptors expressed on the NK cells [82, 83].

Avelumab has been proven in metastatic breast cancer patients. In general, avelumab shows not only a modest antitumor response but also an acceptable safety profile in patients with this disease, especially in the TNBC population, as evidenced by the good tolerability of the evaluated doses and the few reported side effects. In addition, the expression of PD-L1 in TILs identified in breast cancer samples was associated with a better probability of clinical response to avelumab in metastatic breast cancer patients [84]. Different parameters including tumor mutational burden, TIL composition, high microsatellite instability, mismatch-repair deficiency, gut microbiome, and HLA diversity are being evaluated for predicting the therapeutic response to avelumab or other immunotherapies against the PD-1/PD-L1 axis. However, to this date, no clinical or tumor intrinsic-associated parameters have been found; hence, the search for clinical parameters associated with better results still continues [85, 86].

### 4.3. Durvalumab

Durvalumab is also an anti-PD-L1 antibody that has demonstrated clinical efficacy in bladder and lung cancers [87, 88]. The effect of this therapy has not been sufficiently evaluated in patients with TNBC, but the better clinical response is associated with increased stromal TILs (sTILs) and intratumoral TILs (iTILs), in addition to increased pretherapeutic PD-L1 expression on tumor cells. In addition, the administration of durvalumab is associated with the migration of TILs from the stroma toward the tumor cell nests. This increase in the infiltration of immune cells into the tumor was not only evaluated by hematoxylin and eosin staining but was also complemented by a software-assisted standardized approach [89]. Although the increase in the infiltration of immune cells into the tumor did not refer to a specific immune population, it was considered as an indicator of durvalumab response. Nonetheless, the effect of durvalumab is still under investigation, because it has not shown significant changes in pathological complete response when durvalumab is administrated alone or in combination with chemotherapy in early TNBC. Moreover, thyroid dysfunction is a major side effect derived from durvalumab administration [90].

Atezolizumab, durvalumab, and avelumab bind to PD-L1 from various directions and with different binding sites. While atezolizumab binds to the upper side close to the N-terminus of PD-L1, durvalumab and avelumab bind rather perpendicularly to PD-L1. Although any of these antibodies efficiently inhibit PD-1/PD-L1 interaction, they exhibit poor tissue/tumor penetrance due to their large size, which detrimentally affects the therapy. Thus, the development of low-molecular weight proteins or small molecules modulating PD-1/PD-L1 signaling, in addition to their combination with blocking antibodies, might be a promising option for the complete PD-1/PD-L1 blockade in solid tumors [91].

## 5. PD-1 Inhibitors

### 5.1. Pembrolizumab

Pembrolizumab is a humanized anti-PD-1 antibody that has been extensively tested in patients with solid tumors. Nowadays, pembrolizumab is the first-line treatment option for metastatic melanoma and NSCLC. In fact, this drug has been combined with different enzyme inhibitors or chemotherapeutic agents in clinical trials [92, 93]. The use of this drug in cancer treatment has demonstrated promising patient responses with minimal side effects [94]. Pembrolizumab alone has been evaluated in clinical trials in patients with early and metastatic TNBC, showing a better duration of the antitumor response with a pathological complete response (defined as no invasive cancer in the breast and negative nodes) than in patients without treatment. Pembrolizumab has been well tolerated in patients, showing acceptable safety and manageable profile [95–97]. This drug has also been clinically evaluated in patients with early TNBC in combination with neoadjuvant chemotherapy (doxorubicin-cyclophosphamide or epirubicin-cyclophosphamide) or in metastatic TNBC in combination with anthracycline plus taxane therapy, giving a promising antitumor activity with manageable toxicity [98]. In addition, pembrolizumab has also been combined with trastuzumab, which is a monoclonal antibody considered the first-line therapy for breast cancer patients with overexpression of HER2 protein [99]. The combination of two immunotherapies was assessed in HER2+ breast cancer patients resistant to trastuzumab. The addition of pembrolizumab to trastuzumab showed tolerable side effects coupled with a higher percentage of progression-free survival (PFS) as compared with treatment alone in women with advanced disease. It is important to highlight that the authors emphasized that the improvement of this combined therapy was due to the positive expression of PD-L1 on breast cancer cells and TILs [100]. Conversely, the combination of pembrolizumab with chemotherapy (capecitabine/eribulin mesylate) in patients with a luminal breast cancer phenotype does not improve PFS [101, 102]. Thus, further studies are needed to identify molecular biomarkers to select patients who would most likely benefit from PD-1/PD-L1 checkpoint immunotherapies.

On the other hand, similarly to atezolizumab, pembrolizumab has no effects in patients with elevated serum LDH levels, visceral disease, and a high number of metastatic sites in particular in the liver [96, 97]. The aforementioned phenomenon, possibly attributed to a reduced T CD8+ cell density into the tumor, has also been observed with the use of pembrolizumab in other types of neoplasms [103]. Pembrolizumab has also been combined with radiotherapy in metastatic TNBC patients (NCT02730130). At the moment, however, potential biological markers associated with therapeutic success of the clinical efficacy of pembrolizumab alone or in combination have not been found [104].

### 5.2. Nivolumab

Nivolumab is an anti-PD-1 antibody widely studied in the therapeutic practice for patients with melanoma or with metastatic NSCLC after failure of prior chemotherapy based on platinated compounds. Nivolumab treatment has exhibited different side effects after its administration in patients with NSCLC [105]. However, the clinical outcome of nivolumab seems to be good, even after the interruption of its administration in patients with NSCLC. Different comorbidities, such as hypertension and diabetes, have also been associated with patients receiving the PD-1 inhibitor [106]. In patients with TNBC, clinical studies are still ongoing. The effect of nivolumab plus ipilimumab (an anticytotoxic T-lymphocyte antigen 4 (CTLA-4) antibody) vs. nivolumab alone has been evaluated in patients with TNBC. In this study, the combined treatment showed better antitumor results than the treatment alone (NCT01928394). However, a greater toxicity also has been observed after the coadministration. Additionally, nivolumab has also been combined with cabozantinib (a small molecule inhibitor of the tyrosine kinases c-Met and VEGFR2) in treating patients with metastatic TNBC. Nevertheless, the results to date remain inconclusive (NCT03316586). At present, studies about the combination of nivolumab with radiotherapy are also under clinical investigation in patients with metastatic TNBC (NCT02499367). In the same way as the aforementioned PD-1/PD-L1 therapies, no new biological targets that limit or favor the use of nivolumab in breast cancer have been documented.

As we have been mentioning, the response to this kind of biological drug is better and widely studied in TNBC. Much of the differences have been attributed to the status of ER and HER2 expression, high tumor grade, the diversity of tumor-associated immune cells, and PD-1/PD-L1 expression. However, some studies have postulated that the selection of the patients that achieve a successful response to these drugs might fail because of methodological errors, antibody brand, or antibody clone [67, 107, 108]. In this regard, a comparison between three different antibodies (Ventana SP263 by Roche, Dako (22C3) pharmDx antibody, and Biocare Medical RbMCAL 10 antibody) was carried out in different biopsies derived from TNBC patients. This study describes that, although there were few discrepancies among the evaluations of PD-L1 expression in tumor cells using these clones, PD-L1 expression varied not only among tumor types but within a single tumor type at various cutoffs established by the pathologists [108]. In addition, another work also evaluated different clones of anti-PD-L1 antibodies. The authors concluded that detection of PD-L1 in TNBC cells and infiltrated immune cells from biopsies depended on the antibody clone; they found that the amount of staining was highest when using clone E1L3N, followed by clone 28-8 and clone SP142 [109]. Thus, biomarkers that reliably detect PD-L1 expression in breast cancer will increase the benefit for checkpoint immunotherapy.

## 6. Epithelial-Mesenchymal Transition Factors That Determine the Efficacy of PD-1/PD-L1 Therapies in Breast Cancer

**Figure 1:**
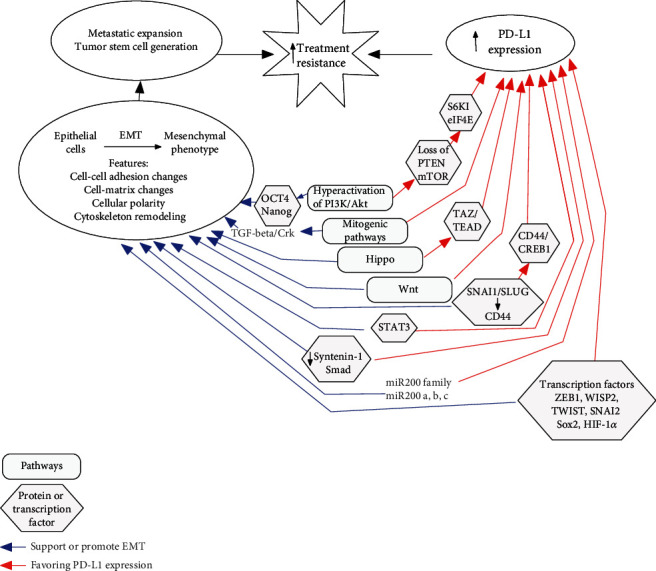
Signaling pathways and transcription factors that interconnect EMT and PD-L1 expression causing increase in PD-L1 expression and consequently failure of immune therapy in breast cancer.

To our knowledge, there is not a compendium of information about the biological molecules that determine the effectiveness of PD-1/PD-L1 therapies in breast cancer patients. Many studies have focused on the regulation of expression of PD-L1 in cancer. Among the mechanisms proposed are proinflammatory cytokines such as IFN-*γ* and genomic and epigenetic alterations [23]. Here, we will discuss molecular factors associated with epithelial-mesenchymal transition (EMT) that might determine the effectiveness and degree of response to PD-1/PD-L1 therapies (Figure 1).

The epithelial-mesenchymal transition (EMT) is a highly dynamic process by which epithelial cells can change their characteristics into a mesenchymal phenotype. The activation of EMT supports tumor progression and metastatic expansion, as well as the generation of tumor stem cell phenotype, which play a major role in resistance to cancer treatment. Generally, EMT comprises the disruption of cell-cell adhesion, cellular polarity, remodeling of the cytoskeleton, and changes in cell-matrix adhesion [110]. It is known that not only tumor cells participate in favoring EMT, but also tumor-infiltrating immune cells, through the secretion of different soluble factors (transforming growth factor- (TGF-) *β*, IL-6, TNF-*α*, CCL18, IFN-*γ*, VEGF, among others), have a positive feedback in the promotion of this process in cancer [111]. Interestingly, several pathways that have been implicated in EMT are also involved in PD-L1 upregulation.

The hyperactivation of the PI3K/AKT pathway has also been linked with the induction of EMT in human mammary cancer cells and the upregulation of PD-L1, in addition to the expression of a well-known stem cell marker: the cluster of differentiation 44 (CD44) molecule [112]. The PI3K/AKT pathway induces PD-L1 at several levels, involving different downstream signaling pathways. The loss of two important factors of the PI3K/AKT pathway, phosphatase and tensin homolog (PTEN) and mammalian target of rapamycin (mTOR), drives the induction of PD-L1 protein levels in breast cancer cells, as well as tumor samples from breast cancer patients. The mechanism is through modulation of downstream proteins, such as S6K1 and eIF4E. In addition, deregulation of PTEN in breast cancer cells increases T cell apoptosis in cocultures, favoring the immune escape by tumor cells [113]. Of note, the activation of PI3K/AKT in breast cancer cells overexpressing PD-1/PD-L1 promotes the expression of two embryonic stem cell transcriptional factors OCT4 and Nanog, which are also associated with the cancer stem cell phenotype [114]. Alternatively, different mitogenic pathways, mainly activated by growth factor receptors, induce adaptor proteins such as Crk, which in turn stabilize the active state of many receptors with or without tyrosine kinase activity through its SH2 and SH3 domains [115, 116]. Interestingly, Crk and TGF-*β* form a positive loop that promotes EMT in lung and breast cancer cells [117], see Figure 1. Cancer cells might evade antitumor immunity through EMT activation together with PD-L1 upregulation [118]. Accordingly, increased expression of Crk in cancer cells has also been associated with high PD-L1 expression in breast cancer cells; the above was observed in a syngeneic mouse model using 4T1 cells [119].

The Hippo signaling pathway has also emerged as a central player in regulating many aspects of tumor biology including higher expression of oncogenes, promotion of EMT, and metastasis [120]. Accordingly, aberrant expression of transcription coactivator 1 (TAZ), a final transducer effector activated through the Hippo pathway, has recently been linked with the boost of PD-L1 levels in human breast cancer cells. The induction of PD-L1 expression was mediated by TAZ through the activation of the TEAD transcription factors with the consequent binding to the PD-L1 promoter. The activation of TAZ was also able to suppress T cell function and induce T cell apoptosis. The above was observed in cocultured TAZ-overexpressing tumor cell lines with Jurkat T cells previously activated with PMA (phorbol 12-myristate 13-acetate) plus PHA (phytohemagglutinin) [121]. Interestingly, the nuclear expression of TAZ has been associated with the TN phenotype in breast cancer [122].

Within the pathways that have also been linked to promoting EMT status is the WNT pathway [123]. Recently, TNBC stem cells were shown to constitutively upregulate PD-L1 through the activation of the WNT signaling pathway. Interestingly, a strong interplay between different negative regulators and final effectors of the WNT pathway together with PD-L1 expression was identified; such association was reversed using WNT inhibitors [124]. Notably, an interaction between TNBC stem cells overexpressing PD-L1 protein with T cells was found using confocal microscopy analysis, suggesting the presence of ineffective antitumor immunity mediated by the activation of the WNT pathway in TNBC stem cells [124]. However, the effectiveness of the T cell response was not evaluated.

Another important protein related to the promotion of the cancer stem cell phenotype is CD44, a transmembrane glycoprotein that is considered a homing cell adhesion molecule. CD44 plays key roles in cell proliferation, motility, and survival, being closely associated with the promotion of the EMT process [125]. The expression of CD44 in breast cancer cells can be upregulated through the activation of different EMT-related transcription factors including SNAI1 and SLUG [126]. CD44 also participates in the switch on different pathways (AKT, STAT3, *β*-catenin, among others) that activate, as a final event, different mesenchymal markers promoting invasion and metastasis in breast cancer cells [127–129]. Of note, the aberrant expression of CD44 has been reported in breast cancer cells, especially in the TN phenotype, conferring poor outcome in the clinic [130, 131]. In addition, CD44 has recently been linked with the increased expression of PD-L1 in TNBC cells. The above is mediated through transcriptional regulation of PD-L1, due to the recognition and junction of the CAMP responsive element-binding protein 1 (CREB1) transcription factor with CD44. The above indicates that the complex CREB1-CD44 can be recruited into a specific consensus-binding sequence of the promoter of PD-L1, boosting its expression [132]. These data point out to CD44 as a critical therapeutic target to suppress the expression of PD-L1.

Signal transducer and activator of transcription 3 (STAT3) is an oncogene involved in the stem cell-like characteristics, proliferation, metastasis, and chemoresistance of breast cancer cells. In addition, activation of STAT3 can promote EMT in breast cancer cells [133]. STAT3 expressed by tumor cells induces the release of different cytokines and chemokines that interact and regulate immune components of the tumor microenvironment (TME), including T cells, NK cells, and tumor-associated macrophages (TAMs), giving a similar inflammatory response to that presented during the wound-healing process [134]. A positive and direct correlation between the activation of STAT3 and the increased expression of PD-L1 in breast cancer cells has been established in both human cell lines and breast cancer samples, being stronger in TNBC specimens. This phenomenon is independent of the mitogenic or proliferative actions of STAT3 [124, 135]. Thus, the employment of STAT3 inhibitors in combination with PD-L1 blockers can be a useful alternative for breast cancer patients with overexpression of STAT3. This strategy is currently being carried out in clinical trials for colorectal cancer (NCT03647839).

In addition to STAT3, there are many transcription factors and miRNAs that have been closely related with the switch to EMT in breast cancer. A positive correlation between different transcription factors and the miRNA 200 family with a high score for PD-L1 expression has been established in human samples of lung adenocarcinomas and squamous lung cancers. A similar behavior has been observed in breast cancer. Overexpression of different EMT-related transcription factors in breast cancer cells has been associated with a high expression of PD-L1 [136]. Among them, different transcription factors have been reported, including twist-related protein 1 (TWIST), slug (SNAI2), snail (SNAI1), zinc finger (ZEB), sex-determining region Y-box 2 (Sox2), and the Wnt 1-inducible-signaling pathway protein 2 (WISP2) [137]. Interestingly ZEB1, WISP2, and SNAI1 but not SLUG strongly upregulate PD-L1 expression in breast cancer cells. Moreover, different members of the miR200 family (miR200a, miR200b, and miR200c) upregulate PD-L1 expression in breast cancer cells [118]. Thus, components involved with the activation of EMT can act differentially in order to upregulate the expression of PD-L1 in breast cancer cells, see Figure 1.

Hypoxia inducible factor- (HIF-) 1*α* is another factor involved in both the promotion of EMT and the regulation of PD-L1 expression. In fact, PD-L1 has hypoxia-response elements (HRE) located in the proximal PD-L1 promoter; this phenomenon was reported in myeloid-derived suppressor cells (MDSCs) [138]. Moreover, in TNBC cell lines, the increased expression of PD-L1 has also been observed after exposure to hypoxic conditions. The low levels of oxygen in cell cultures also favored apoptosis of T cells after coincubation with breast cancer cells exposed to a hypoxic environment, thus confirming a direct relationship between HIF-1*α* and PD-L1 [139].

Syntenin-1 (syndecan-binding protein, SDCBP), also known as melanoma differentiation-associated gene-9 (MDA9), participates in the induction of the EMT process by positively regulating Smad activation, mediated by TGF-*β*1 [140]. In addition, syntenin-1 also causes the upregulation of the expression of PD-L1 in breast cancer cells [141]. Syntenin-1 is a PDZ domain-containing molecule, which was identified as a key oncogene in melanoma. Moreover, different types of cancer, including glioblastoma; neuroblastoma; and prostate, breast, and liver cancer exhibit aberrant expression of syntenin-1 [142]. Recent findings demonstrated that, through phosphorylation of STAT3, syntenin-1 increases the expression of PD-L1 in both breast cancer cell lines and in tumor tissues derived from patients with TNBC cocultured with T CD8+ cells. Thus, syntenin-1 induces CD8+ T cell apoptosis *in vitro* and *in vivo* by upregulating PD-L1 [141].

Overall, the mechanisms reported above support the need to look for a pan-cancer EMT signature in patients with TNBC, which would lead to implement current inhibitors for the EMT players [143] in order to warrant the success of PD-1/PD-L1 therapies.

## 7. Metabolism-Associated Mechanisms Support PD-1/PD-L1 Signaling Favoring Tumoral Escape

**Figure 2:**
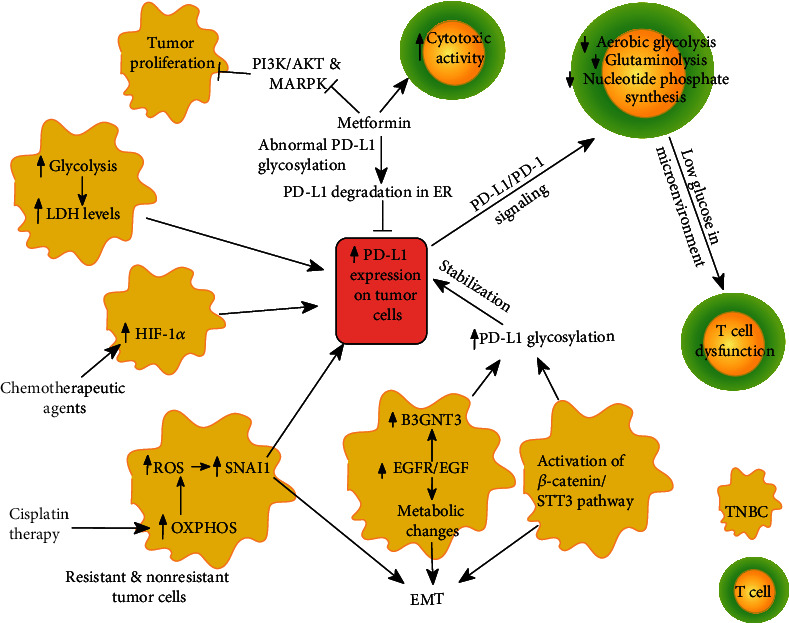
Metabolism-associated mechanisms supporting PD-L1 expression in breast cancer tumors.

In clinical studies, strong PD-L1 expression has been significantly associated with increased risk of recurrence along with increased uptake of a glucose analogue (18F-fluorodeoxyglucose) in TNBC patients [144]. However, the relationship between increased glycolysis and the expression of PD-L1 in TNBC tumors is not clear [144]. Conversely, different PD-1/PD-L1 therapies have been reported to be ineffective against breast tumors with increased serum LDH levels (Figure 2). Therefore, modulation of the environment triggered by tumor reprogramming affects the clinical outcome of PD-1/PD-L1 therapies [22, 76, 96, 97].

An outstanding finding involving metabolic changes is that PD-1/PD-L1 signaling can shift T cell metabolism away from aerobic glycolysis and glutaminolysis, reducing the glycolytic intermediates, as well as consumption of glutamine (Figure 2). Moreover, activation of the PD-1/PD-L1 pathway forces the T cell to utilize alternative substrates to feed the tricarboxylic acid (TCA) cycle. In addition, the engagement of PD-L1 with its receptor blocks the nucleoside phosphate de novo synthesis in T cells [145]. The above has been observed by sophisticated metabolomic assays, where human PBMCs were cultured to expand an activate the T cell lineage and further treated with a recombinant human Fc-tagged PD-L1 fragment. Finally, different metabolites were obtained and analyzed by mass spectroscopy in order to offer a metabolite profile encompassing around 155 polar and semipolar metabolites [145]. Thus, several authors have postulated that it may be advantageous to conjugate immune-checkpoint blockade with metabolic interventions. In fact, high glucose consumption together with competition for key amino acids by tumor cells can leave T cells with insufficient energy and biosynthetic precursors to support activities, such as cytokine secretion. Thus, T cells are rendered dysfunctional [146]. A futuristic view could also include metabolic reinforcement with the help of cell engineering in cytotoxic T cells, such as chimeric antigen receptor T cells (CAR T cells). Thus, metabolic engineering of CAR T cells alongside checkpoint immunotherapy might improve clinical outcomes in breast cancer patients.

Posttranslational modification of PD-L1 such as glycosylation, phosphorylation, ubiquitination, deubiquitination, and lysosomal degradation supports the generation of resistance to PD-L1 therapies [147]. For instance, glycosylation of PD-L1 protects the protein from degradation in stem, as well as non-stem breast cancer cells, suppressing the cytotoxic activity of T cells [148]. One of the mechanisms described, by which the glycosylation of PD-L1 can be achieved in breast cancer cells, involves the activation of the epidermal growth factor receptor (EGFR) signaling through binding of the epidermal growth factor (EGF) and the concomitant overactivation of the *β*-1,3-N-acetylglucosaminyl transferase (B3GNT3) [149]. The aforementioned results suggest that EGFR signaling participates not only in the metabolic changes in breast cancer cells but also in their promotion into an EMT phenotype as it has been already reported. The latter study reinforces the idea that EGFR signaling not only promotes the EMT phenotype but also participates in the metabolic changes in breast cancer cells [150]. Another mechanism that supports the glycosylation of PD-L1 in breast cancer stem cells is the activation of the EMT/*β*-catenin/STT3/PD-L1 signaling axis. Interestingly, the activation of this axis activates N-glycosyltransferase STT3, the enzyme responsible for this posttranslational modification, which leads to subsequent PD-L1 stabilization, upregulation, and consequent immune evasion [151]. Of note, STT3 is also closely associated with the maintenance of the EMT process [152].

Thus, we propose that the combination of EMT inhibitors with antibodies against glycosylated forms of PD-L1 will improve the therapeutic outcome of PD-1/PD-L1 therapies in patients with breast cancer, specifically with the TN phenotype.

Additionally, some drugs can affect both the cell metabolism and the expression of PD-L1. Metformin, a drug linked with the regulation of the glucose metabolism in patients with diabetes type 2, has been shown to impulse the tumor surveillance through the maintenance of induction of effector/memory CD8+ T cells [153]. Metformin reduces the proliferation of tumor cells by inhibiting PI3K/AKT and MAPK pathways [154]. Metformin also decreases PD-L1 levels through inducing abnormal PD-L1 glycosylation with Man9GlcNAc2, a rare glycan structure, which results in its degradation in the endoplasmic reticulum in breast cancer cells [155–157], see Figure 2. In addition, metformin regulates the secretion of different proinflammatory cytokines, including IL-1*β*, IL-6, IFN-*γ*, and TNF-*α*. This phenomenon leads to the attenuation of the inflammatory process in cancer, thus promoting the antitumor immune response [156, 158, 159]. Hence, metformin might be used as a new regulator of the expression of PD-L1 in combination with PD-1/PD-L1 target therapies.

It is widely known that TNBC tumors lack a target therapy. For this reason, they are treated with cytotoxic therapy [160]. In this sense, the neoadjuvant chemotherapy (NAC) based on cisplatin also induces resistance in TNBC [161]. Interestingly, the conventional treatment of cisplatin in NSCLC has been shown to induce drug resistance through the alteration of tumor metabolism [162]. Therefore, cisplatin-resistant cells become more reliant on mitochondria oxidative metabolism mediated by the production of reactive oxygen species (ROS) instead of glucose metabolism [163, 164]. On the other hand, high ROS levels, along with metabolic alterations, contribute to the EMT process through the activation of the transcription factor SNAI1 in breast cancer cells [165] (Figure 2). Treatment with cisplatin and the subsequent generation of cisplatin-resistant breast cancer cells might also induce PD-L1 expression in breast cancer; nonetheless, this statement has not been fully addressed [166].

Interestingly, NAC not only may impact on the metabolic pathways of cancer cells, but it may also affect the number of immune cells recruited into the tumor microenvironment or in circulation [167]. For instance, in breast cancer patients with the TN phenotype, the administration of NAC based on anthracycline or taxane modified the infiltration of the neutrophil ratio [168], which has been associated with a proinflammatory profile that favors poor patient survival outcomes in early and advanced TNBC [169]. Nonetheless, unconvincing results about the modulation of the expression of PD-L1 in the breast cancer cells were reported [168].

Other chemotherapeutic agents including carboplatin, doxorubicin, gemcitabine, or paclitaxel have also been shown to induce the expression of PD-L1 in TNBC cells through a HIF-1*α*-dependent transcriptional mechanism [170], see Figure 2. In addition, coculture of breast cancer cells, treated with these chemotherapeutic drugs, with activated T CD8+ cells under hypoxic conditions, results in the inhibition of T cell activity and CD8+ T cell apoptosis [170]. This again suggests that chemotherapy induces important metabolic changes that impact in the expression of PD-L1, which eventually would lead to the evasion of the immune system.

## 8. Conclusion

We have reviewed pathways, transcription factors, as well as molecules that interconnect EMT with PD-L1 expression, hence supporting treatment resistance in TNBC. Among the variables that should be taken into account for increasing the success of antitumor therapies based on immune checkpoints, metabolism-associated mechanisms promoting PD-L1 expression, alongside with postranslational mechanisms that stabilize PD-L1 structure, must be considered. We suggest that PD-1/PD-L1 checkpoint therapies could be greatly improved in TNBC with the employment of inhibitors of EMT and metabolic reprogramming of CAR T cell therapies, as well as the avoidance of the combination of PD-1/PD-L1 therapies with chemotherapeutic agents. The use of mathematical models that incorporate tumor-immune cell dynamics might provide quantitative representations of the phenomena involved in cancer progression [171]. This approach would be useful to mimic molecular networks and to search for novel networks. Consequently, a more comprehensive knowledge would be obtained concerning the interaction among EMT, metabolic reprogramming, epigenetic modifications, and drug interactions, which, all together, govern PD-L1 expression in breast cancer.

## Figures and Tables

**Table 1:** Types of PD-1/PD-L1 inhibitors tested in clinical trials mostly evaluated in breast cancer patients with TN phenotype.

Inhibitor	Population of study	Treatment scheme	Outstanding results/response rates to therapy	References
PD-L1 inhibitors				
Atezolizumab	Metastatic TNBC (mTNBC) patients	One or more doses of atezolizumab (840 mg) combined with nanoparticle albumin-bound- (nab-) paclitaxel	Antitumoral responses were denoted by the change in tumor burden The progression-free survival (PFS) was 8.6 months with the combination of atezolizumab plus nab-paclitaxel vs. 5.1 months with the paclitaxel alone The objective response rate (ORR) was 53.8% Overall survival (OS) was 24.2 months with the combined treatment vs. 12.4 months with paclitaxel alone No serious side effects were reported	[75]
Atezolizumab	Patients with advanced mTNBC	Intravenous atezolizumab (840 mg) combined with nab-paclitaxel (100 mg/m^2^) on days 1, 8, and 15 of every 28-day cycle	The combined treatment increased PFS in patients with mTNBC PFS was 7.2 months with atezolizumab plus nab-paclitaxel as compared with 5.5 months with nab-paclitaxel alone OS was 21.3 months with atezolizumab plus nab-paclitaxel and 17.6 months with placebo plus nab-paclitaxel No serious side effects were reported	[77]
Avelumab	Patients with locally advanced or metastatic breast cancer	Intravenous avelumab (20 mg/kg) every 2 weeks for 10 months approximately	The study compared patients with breast cancer of diverse phenotypes The ORR of patients with the TN phenotype was 5.2% versus 2.8% found in patients with HER2-/ER/PR+ phenotype Additionally, the rate of PFS was higher in the TN population (12.4%) than in the hormonal counterpart (1.01%). However, the OS rate at 12 months was lower in patients with the TN phenotype, being 37.1% vs. 40.3% in the other phenotype Patients presented moderate side effects accompanied with grade 3 side effects in some patients	[84]
Durvalumab	Patients with primary non-metastatic-TNBC	Monotherapy of intravenous durvalumab (0.75 g) 2 weeks before start of standard neoadjuvant chemotherapy (NACT) based on nab-paclitaxel followed by dose-epirubicin/cyclophosphamide (EC) Following of intravenous durvalumab 1.5 g every 4 weeks plus nab-paclitaxel 125 mg/m^2^ weekly for 12 weeks	Increased stromal TILs (sTILs), intratumoral TILs (iTILs), and increased pretherapeutic PD-L1 expression in tumor cells Pathological complete response (pCR) rate with durvalumab plus NACT was 58% vs. placebo 44.4% Importantly, patients presented thyroid dysfunction as a major side effect	[90]
PD-1 inhibitors				
Pembrolizumab	Patients with early TNBC	Pembrolizumab alone (200 mg) or pembrolizumab plus, paclitaxel and carboplatin for 3 weeks. Both groups were later complemented with doxorubicin-cyclophosphamide or epirubicin-cyclophosphamide	Side effect was grade 3 The pCR was 64.8% in the pembrolizumab-chemotherapy and 51.2% in the placebo-chemotherapy group Patients showed side effects grade 3	[95]
Pembrolizumab	Patients with mTNBC	Intravenous pembrolizumab (200 mg) every 3 weeks for up to 2 years	The activity of pembrolizumab was compared between PD-L1-positive and PD-L1-negative women The median OS was 9.0 months in all patients The ORR of the pembrolizumab monotherapy for PD-L1-positive tumors was 5.7% vs. 4.7% for patients with PD-L1-negative tumors The PFS for PD-L1-positive tumors were 8.7% vs. 7.3% in PD-L1-negative tumors Pembrolizumab monotherapy demonstrated durable antitumor activity in patients with mTNBC There were no responses in patients with liver metastases Side effects on some patients were grades 3 and 4	[96]
Pembrolizumab	Patients with advanced TNBC	Single-agent pembrolizumab given intravenously at 10 mg/kg every 2 weeks until completing 36 doses	The patients experienced a decrease from baseline in tumor burden and the decrease was maintained during all of the time of study Some patients showed an increase in the serum levels of LDH which was associated with the poor response of pembrolizumab The overall response rate was 18.5% The major side effects found in patients were arthralgia, fatigue, myalgia, and nausea and some grade 3 side effects	[97]
Pembrolizumab	Patients in early-stage TNBC	Intravenous pembrolizumab (200 mg) plus chemotherapy (with taxane with or without carboplatin) for 12 weeks and then doxorubicin and cyclophosphamide for an additional 12 weeks before surgery (6 different cohorts)	The number of TILs were higher in patients with a positive expression of PD-L1 The study showed promising antitumor activity as demonstrated by the high pCR rates around 60% across all cohorts The overall survival in all of the cohorts was around 10-18% higher when a combined treatment was administrated compared with treatment alone Neutropenia was the most common side effect	[98]
Pembrolizumab	Patients with advanced, unresectable, or metastatic HER2-positive breast cancer and previous resistance to trastuzumab	Intravenous pembrolizumab (2 mg/kg and 10 mg/kg, every 3 weeks) plus 6 mg/kg of intravenous trastuzumab every 3 weeks (1 cycle) until 35 cycles were reached	The combined treatment in the PD-L1-positive cohort had the following results: PFS was 6-12-months; the PD-L1 negative cohort had 2.5 months of PFS. However, there were no objective responses, and no patient achieved control of the disease Some patients showed grade 3 to 5 side effects. Neutropenia was the most common side effect	[100]
Pembrolizumab	Patients with TN and hormone receptor (HR) positive/HER2 negative endocrine-refractory metastatic breast cancer	Intravenous pembrolizumab (200 mg) and capecitabine (1000 mg/m^2^) on days 1–14 of a 21-day cycle	The study compared the PFS and OS rates between patients with TN and ER+ phenotypes The median PSF and the median OS in TN patients were 4 and 15.3 months, respectively, vs. 5.1 months and were not significantly different compared with the hormone-positive phenotype The study also evaluated the effects of the combined treatment in patients with PD-L1-positive or PD-L1 negative expression. The clinical benefit rate was 33.3% in the PD-L1-positive population vs. 22.32% found in the negative counterpart Most common adverse events were low grade and consistent with those seen in patients with a metastatic disease that received capecitabine alone, including hand-foot syndrome, gastrointestinal symptoms, fatigue, and cytopenia	[101]
Pembrolizumab	Patients with HR+/HER2- metastatic breast cancer	Intravenous pembrolizumab (200 mg/m^2^) epirubicin (1.4 mg/m^2^) intravenously	The PFS and ORR were not different between the patients that received the combined treatment or the treatment alone, being PFS 4.1 vs. 4.2 months, respectively, and the ORR 25% *vs*. 34%, respectively. Of note, all of patients were PD-L1 positive The combination of epirubicin and pembrolizumab was not associated with longer PFS compared with epirubicin alone	[102]
Pembrolizumab	Patients with mTNBC patients	Intravenous pembrolizumab (200 mg) combined with radiotherapy (3000 cGy).	The studies are ongoing	NCT02730130
Nivolumab	Patients with TNBC	Intravenous nivolumab (1 mg/kg) plus ipilimumab (1 mg/kg) every 3 weeks for 4 doses followed by nivolumab 3 mg/kg every 2 weeks until documented disease progression	The studies are ongoing, although better antitumor results than the treatment alone has been observed	NCT01928394
Nivolumab	Patients with TNBC	Intravenous nivolumab given every 4 weeks and cabozantinib given orally once daily	The studies are ongoing	NCT03316586
Nivolumab	Patients with mTNBC	Each patient was administered with intravenous nivolumab (3 mg/kg) plus radiotherapy (20 Gy to metastatic lesion) doxorrubicin (15 mg) once weekly for 2 weeks, cyclophosphamide (50 mg) daily, and cisplatin (40 mg/m^2^) weekly, respectively, for 2 weeks	The studies are ongoing	NCT02499367

mTN: metastatic triple negative; ORR: objective response rate; OS: overall survival; pCR: pathological complete response; PFS: progression-free survival; TN: triple negative; TNBC: triple-negative breast cancer.

## Data Availability

No data were used to support this study.
